# Identification of MAD2L1 as a novel biomarker for hepatoblastoma through bioinformatics and machine learning approaches

**DOI:** 10.3389/fonc.2025.1524714

**Published:** 2025-03-31

**Authors:** Ying He, Xiwei Hao, Bin Hu, Nan Xia, Chaojin Wang, Xin Chen, Huanyu Zhang, Yuhe Duan, Qinglong Ying, Qian Dong

**Affiliations:** ^1^ Department of Pediatric Surgery, The Affiliated Hospital of Qingdao University, Qingdao, China; ^2^ Department of Radiology, The Affiliated Hospital of Qingdao University, Qingdao, China; ^3^ Shandong Key Laboratory of Digital Medicine and Computer Assisted Surgery, The Affiliated Hospital of Qingdao University, Qingdao, China; ^4^ Shandong College Collaborative Innovation Center of Digital Medicine Clinical Treatment and Nutrition Health, Qingdao University, Qingdao, China

**Keywords:** hepatoblastoma, biomarkers, WGCNA, machine learning, MAD2L1

## Abstract

**Objective:**

This study aims to identify potential biomarkers for Hepatoblastoma (HB) using bioinformatics and machine learning, and to explore their underlying mechanisms of action.

**Methods:**

We analyzed the datasets GSE131329 and GSE133039 to perform differential gene expression analysis. Single-sample gene set enrichment analysis (ssGSEA) and weighted gene co-expression network analysis (WGCNA) were utilized to identify gene modules linked to gene set activity. Protein-protein interaction (PPI) networks were constructed to identify hub genes, while random forest and support vector machine models were employed to screen for key diagnostic genes. Survival and immune infiltration analyses were conducted to assess the prognostic significance of these genes. Additionally, the expression levels, biological functions, and mechanisms of action of the selected genes were validated in HB cells through relevant experimental assays.

**Results:**

We identified 1,377 and 1,216 differentially expressed genes in datasets GSE131329 and GSE133039, respectively. ssGSEA and WGCNA analyses identified 234 genes significantly linked to gene set activity. PPI analysis identified 20 core Hub genes. Machine learning highlighted three key diagnostic genes: CDK1, CCNA2, and MAD2L1. Studies have demonstrated that MAD2L1 is significantly overexpressed in HB and is associated with prognosis. WGCNA revealed that MAD2L1 is enriched in gene sets related to E2F_ TARGETS and G2M_CHECKPOINT. Experimental assays demonstrated that MAD2L1 knockdown significantly inhibits the proliferation, migration, and invasion of HB cell lines, and that MAD2L1 promotes cell cycle progression through the regulation of E2F.

**Conclusion:**

Our study identifies MAD2L1 as a novel potential biomarker for HB, providing new strategies for early diagnosis and targeted therapy in HB.

## Introduction

Hepatoblastoma (HB) is a prevalent primary malignant liver tumor in children, occurring more frequently in males than in females (1), accounting for approximately 80% of pediatric liver tumors (2). The most effective treatment for pediatric HB is surgical resection. However, due to the often-extensive liver involvement in children, complete surgical removal is frequently not possible. Consequently, current clinical management primarily involves a combination of surgical resection and chemoradiotherapy (3). In all diagnosed patients, systemic chemotherapy and surgery can lead to a 5-year survival rate of 80% in children (4). Despite advancements, the prognosis for HB patients remains suboptimal, largely due to the absence of effective early diagnostic methods. Additionally, prolonged treatment often leads to various complications and side effects (5, 6). Currently, clinicians primarily rely on clinical symptoms, imaging features, and alpha-fetoprotein levels to diagnose HB. However, AFP levels can be elevated due to numerous conditions in pediatric patients, resulting in insufficient sensitivity and specificity, thus highlighting the inadequacy of early diagnostic methods (7). Moreover, HB often presents insidiously, with complex influencing factors and limited sample sizes, leaving its pathogenesis insufficiently elucidated. Consequently, new reliable diagnostic methods and biomarkers beyond AFP are needed to support timely diagnosis and optimal treatment, thereby improving prognostic accuracy.

Recent advances in high-throughput sequencing and microarray analysis have facilitated the identification of disease-related biomarkers and the study of gene functions and pathways (8, 9). By integrating bioinformatics and machine learning, researchers can effectively discover tumor biomarkers across various cancers (10–14). Studies have shown that the mRNA that is abnormally expressed in HB can be identified by bioinformatics methods (15–17). Weighted Gene Co-Expression Network Analysis (WGCNA), an unsupervised method, is widely used in bioinformatics to explore inter-gene relationships in high-throughput data. WGCNA constructs weighted co-expression networks to identify gene modules that reflect specific biological processes (18).

In this study, based on bioinformatics and machine learning methods, diagnostic and prognostic biomarkers of hepatoblastoma were identified, and biological functions and mechanisms of action of key genes were validated through *in vitro* experiments. In our study, we analyzed the association between the gene set activity and HB gene expression pattern for the first time, thereby revealing the potential regulatory networks and molecular mechanisms.

## Materials and methods

### Identification of Differentially Expressed Genes (DEGs)

We downloaded raw data from the GEO database (GSE131329 and GSE133039). GSE131329 (Affymetrix GPL6244) contains 53 HB and 14 non-cancerous liver samples, while GSE133039 (Illumina GPL16791) includes 31 tumors and 32 non-cancerous samples. After the data is preprocessed (**Supplementary Material**), GSE131329 was used for training and GSE133039 for validation. We used the Limma package for microarray data and DESeq2 for sequencing data, identifying DEGs with |Log_2_FC| > 1 and P < 0.05 (19, 20).

### Weighted Gene Co-Expression Network Analysis (WGCNA)

We employed single-sample gene set enrichment analysis (ssGSEA) to calculate the enrichment scores of classical gene sets in each sample. The gene set used was derived from the Hallmark gene set in the Broad Institute’s MSigDB database (h.all.v2023.1.Hs.symbols.gmt). Differences between normal and tumor samples were assessed using the Wilcoxon rank-sum test.

To enhance the sensitivity of WGCNA, we selected the top 10,000 genes with the highest variability based on the Median Absolute Deviation (21). Hierarchical clustering was performed to identify and remove outliers, resulting in stable outcomes visualized in a clustering tree. We calculated network attributes at various soft thresholds to determine the optimal threshold for constructing a scale-free co-expression network. The adjacency and topological overlap matrices facilitated the identification of gene modules. Using a dynamic tree cutting method, we identified gene modules and calculated their feature vectors. High correlations (>0.75) among color modules prompted their merger to simplify the network structure (22). Finally, we analyzed the correlation between gene expression patterns and gene module feature vectors to determine the relationship between gene modules and gene set enrichment scores. Gene modules with a correlation to the enrichment score greater than 0.8 and a P-value less than 0.05 were selected for subsequent analyses.

### Protein-Protein Interaction (PPI) network construction of hub genes and enrichment analysis

We constructed PPIs using the STRING online database (23), setting an interaction score threshold of 0.4 for network reliability. The PPI network was visualized using Cytoscape software (24), where key genes were identified through Cytoscape plug-ins (25). To determine biologically significant key genes, we calculated each node’s degree value and selected the top 20 hub genes using the CytoHubba plug-in. Subsequently, we performed Gene Ontology (GO) and Kyoto Encyclopedia of Genes and Genomes (KEGG) enrichment analysis on these key genes. This analysis revealed potential biological functions and signaling pathways. GO analysis encompasses three areas: Biological Process, Molecular Function, and Cellular Component, while KEGG is a recognized database that identifies gene interactions and their roles in biological systems.

### Machine learning algorithms and ROC curves

Random forest (RF) is an ensemble learning method that enhances classification by constructing multiple decision trees and aggregating their predictions. By randomly selecting data and feature subsets, RF improves accuracy and mitigates overfitting through majority voting (26). In this study, we optimized the number of trees by analyzing the out-of-bag error curve, selecting the configuration that minimized the classification error. To evaluate feature importance, we computed the Mean Decrease in Gini Index, which measures the contribution of each gene to reducing classification impurity. We ranked genes based on their importance scores and identified the top five key biomarkers for HB classification, visualizing their relative significance through importance plots.

Support vector machine recursive feature elimination (SVM-RFE) is a feature selection technique that iteratively removes the least informative features based on a trained support vector machine (SVM) model. By recursively eliminating features with the smallest contribution to classification performance, SVM-RFE improves model generalization and reduces overfitting (27, 28). To ensure robust feature selection, we applied 10-fold cross-validation to evaluate the model performance at each iteration, plotting error rate curves to illustrate the impact of feature count on classification accuracy. The combination of RF and SVM-RFE allowed us to identify a refined set of features that contribute most significantly to HB classification, improving both model interpretability and predictive accuracy.

### Survival analysis and immune infiltration analysis

To explore whether key genes can be used as a prognostic biomarker for HB, we used the GEPIA database (Gene Expression Profiling Interactive Analysis, http://gepia.cancer-pku.cn/) to generate Kaplan-Meier survival curves. To explore how key gene expression patterns influence immune cell distribution in the tumor microenvironment, we utilized the ssGSEA method to analyze the infiltration of 24 immune cell types in tumor samples (29). We compared immune cell infiltration between high and low expression groups of key genes to assess correlations with expression levels (30, 31). Spearman correlation analysis and the Wilcoxon rank sum test were conducted to detect differences in immune cell infiltration.

### Clinical sample collection

This study included samples from 6 patients who underwent radical surgery for hepatoblastoma in the pediatric surgery department of the affiliated hospital of Qingdao University from 01/08/2018 to 30/12/2023, covering tumor tissues and adjacent tissues. The specimens obtained during the operation were placed in a freezing storage tube in accordance with standard methods and stored in a refrigerator at -80°C in the department of pathology. The study was approved by the Ethics Committee of the Affiliated Hospital of Qingdao University (Ethics number: QYFY, WZLL28988) and informed consent of all participating patients’ parents or guardians was obtained. The study was conducted in accordance with the declaration of Helsinki. The tumor tissues and adjacent tissues from the affiliated hospital of Qingdao university for the study were accessed for our study on 07/08/2024.

### Cell culture

The HuH6 and HepG2 cell lines were obtained from Wuhan Pricella Biotechnology Co., Ltd. HuH6 cells were grown in DMEM supplemented with 10% fetal bovine serum (FBS, Cat. No. C04001-050, VivaCell) and 1% penicillin-streptomycin (Cat. No. C3420-0100, VivaCell). HepG2 cells were maintained in MEM with the same concentrations of FBS and antibiotics (Cat. No. PM150410, Procella). Both cell lines were incubated under optimal growth conditions at 37°C and 5% CO_2_.

### CCK-8 assay

During logarithmic growth, cells were digested 3,000 cells per well were seeded in 96-well plates. After adherence, 100 μ0 of medium with 10% CCK8 (Cat. No. HY-K0301, MCE) was added. Cells were incubated at 37°C, 5% CO_2_ (HuH6 for 2 hours, HepG2 for 1 hour), and optical density at 450 nm was measured. Cell viability was assessed over 5 days.

### Colony formation assay

Cells in the logarithmic growth phase were seeded into 6-well plates at a density of 3,000 cells per well. The plates were gently rocked in a figure-eight motion to ensure uniform cell distribution. The plates were then incubated in a cell culture incubator at 37°C with 5% CO2 for 14 days. After the incubation period, the medium was aspirated, and the wells were gently washed three times with PBS (Cat. No. PB180327, Pricella). To fix the colonies, 1 mL of 4% paraformaldehyde (Cat. No. G1072) was added to each well, and the cells were fixed for 20 minutes. After removing the paraformaldehyde, 1 mL of 0.5% crystal violet (Cat. No. BL539A, biosharp) solution was added to each well for 20 minutes to stain the colonies. The crystal violet solution was then removed, and the wells were gently rinsed with a steady flow of water. The plates were air-dried at room temperature. To quantify colony formation, images were captured using a flatbed scanner or under plate lights.

### Transwell invasion assay

Cells in the logarithmic growth phase were digested with trypsin and seeded at a density of 1.5×10^5 cells in a small chamber with a specified pore size 8um. The upper chamber contained DMEM/MEM basal medium without fetal bovine serum (FBS), while the lower chamber was filled with complete DMEM/MEM medium supplemented with 10% FBS and 1% penicillin-streptomycin. After the incubation period, cells were fixed with 4% paraformaldehyde and stained with 0.5% crystal violet to enable subsequent observation and analysis of their invasion capacity.

### Scratch wound healing assay

We employed the Jibidi Culture-Insert 4 Well in a 35 mm u-Dish (Cat. No: 80466) to uniformly seed 2×10^5 cells in each of the four wells. After allowing the cells to adhere, the Culture-Insert was removed. HuH6 cell lines and HepG2 cells were photographed under EVOS fluorescence microscopy at 0 h and 48 h to observe changes in cell migration and repair ability.

### Dual-luciferase reporter assay

Logarithmic growth cells were taken and 100 UL cell suspensions of 3*10^4 cells/well were inoculated on an all-black 96-well cell culture plate (FCP966, Beyotime). Si-NC or Si-MAD2L1 were transfected with siRNA-Mate plus transfection reagent (G04026, GenePharma) 24h, and then use GP-transfect-Mate (G04008 Genepharma) Reporter plasmid pE2F-TA-Luc (D4054, Beyotime) and Reporter plasmid pRL-TK (D2760, Beyotime) was transfected for 48h. The cell culture plate was taken out and balanced at room temperature for 10 minutes, and 100ul Dual-Lumi™ firefly luciferase detection reagent (RG088S, Beyotime) was added to each well, and incubated at room temperature for 10 minutes before detection with a multifunctional enzyme labeler. Then 100ul Dual-Lumi™ sea kidney luciferase assay working solution (RG088S, Beyotime) was added to each well and incubated at room temperature for 10 minutes before detection. The relative luciferase activity was standardized using sea kidney luciferase as the internal reference.

### Flow cytometry assay

Cells were collected, and cell suspension with a final concentration of 1x10^6 cells/ml was prepared, the supernatant was removed by centrifugation, and 1.2ml of anhydrous ethanol preserved at -20°C was added after washing with PBS, and then fully mixed and fixed at -20°C overnight. The supernatant was removed by centrifugation, washed with PBS once, and 100ul of RNase A Reagent was added to fully suspend the cells, and the cells were immersed in 37°C water bath for 30min. Add 400ul of PI Reagent (50ug/ml), incubate at 4°C for 30min without light, test immediately on the computer, and use NovoExpress software for mapping.

### Quantitative real-time PCR analysis (qRT-PCR)

Total RNA was extracted using Trizol reagent (Cat. No. E701-01, Vazyme). cDNA synthesis utilized the 5X ABScript mRNA reverse transcription kit (Cat. No. RM21478) and 20X genomic DNA removal reagent (Cat. No. RM21479, ABclonal). PCR used β-ACTIN as the reference gene (Cat. No. N901r, CellGene) with 2X SYBR Green PCR Master Mix (Cat. No. RM21203, ABclonal). Gene expression was normalized to β-ACTIN and analyzed via the 2-ΔΔCt method. Primer sequences: β-ACTIN (F: GAGAAAATCTGGCACCACACC, R: GGATAGCACAGCCTGGATAGCAA); MAD2L1 (F: ACGGACTCACCTTGCTTGTA, R:CCAGGACCTCACCACTTTCA).

### Western blotting analysis (WB)

HB tissue samples and cell lines were washed twice with PBS and lysed on ice with RIPA buffer (Cat. No. R0010, Solarbio) containing PMSF for 30 minutes. The lysate was centrifuged at 12,000 rpm at 4°C for 10 minutes to collect the supernatant. Tissue protein concentration was measured using the BCA protein assay kit (Cat. No. P0012, Beyotime). Fifty micrograms of protein were separated via 10% SDS-PAGE and transferred to PVDF membranes, which were blocked with 5% skim milk at room temperature for 1 hour. Primary antibodies against MAD2L1 (1:600, Cat. No. 15283-1-AP, Proteintech), β-ACTIN (1:10,000, Cat. No. N901r, CellGene), β-Tubulin (1:1,500, Cat. No. E-AB-40518, Elabscience), GAPDH (1:3000, Cat. No.AF7021, AFFINITY), N-Cadherin Rabbit mAb (1:1000, Cat. No. A19083, ABclonal), E-Cadherin Rabbit mAb (1:1000, Cat. No. A20798, ABclonal), Cyclin A2 Polyclonal antibody (1:10000, Cat. No. 18202-1-AP, Proteintech), PCNA Polyclonal antibody (1:5000, Cat. No. 10205-2-AP, Proteintech), CyclinE1(1:1000, Cat. No. 11554-1-AP, proteintech) and E2F3(1:1000, Cat. No. 27615-1-AP, proteintech) were incubated overnight at 4°C. After rinsing with PBST, membranes were incubated with 1:25,000 diluted goat anti-Rabbit IgG (HRP, ab190495; Abcam) for 1 hour at room temperature. Protein visualization was achieved using the SuperPico ECL Chemiluminescence Kit (Cat. No. E422-01, Vazyme), and quantitative analysis of protein bands relative to β-actin or β-Tubulin was performed using ImageJ software.

### Cell transfection

The siRNAs were transfected into cells using siRNA-Mate plus transfection kit (GenePharma, China) according to the manufacturer’s instructions. The gene changes were detected by qPCR 24 hours after transfection, 48 h after transfection, WB was used to verify the transfection efficiency. The following sequences of siRNAs were used:

siNC: 5′-UUCUCCGAACGUGUCACGUTT-3′.

siMAD2L1-1(655):5′-CCGCCUUCGUUCAUUUACUTT-3′.

siMAD2L1-2(364):5′-GGUUGUAGUUAUCUCAAAUTT-3′.

siMAD2L1-3(260):5′-GGACUCACCUUGCUUGUAATT-3′.

siMAD2L1-4(316):5′-GGUGGAACAACUGAAAGAUTT-3′.

### Statistics

Statistical analysis was performed using R software version 4.3.1 and GraphPad Prism 9.5. P < 0.05 was considered statistically significant.

## Result

### Difference analysis results of GSE131329 and WGCNA

**Figure 1:**
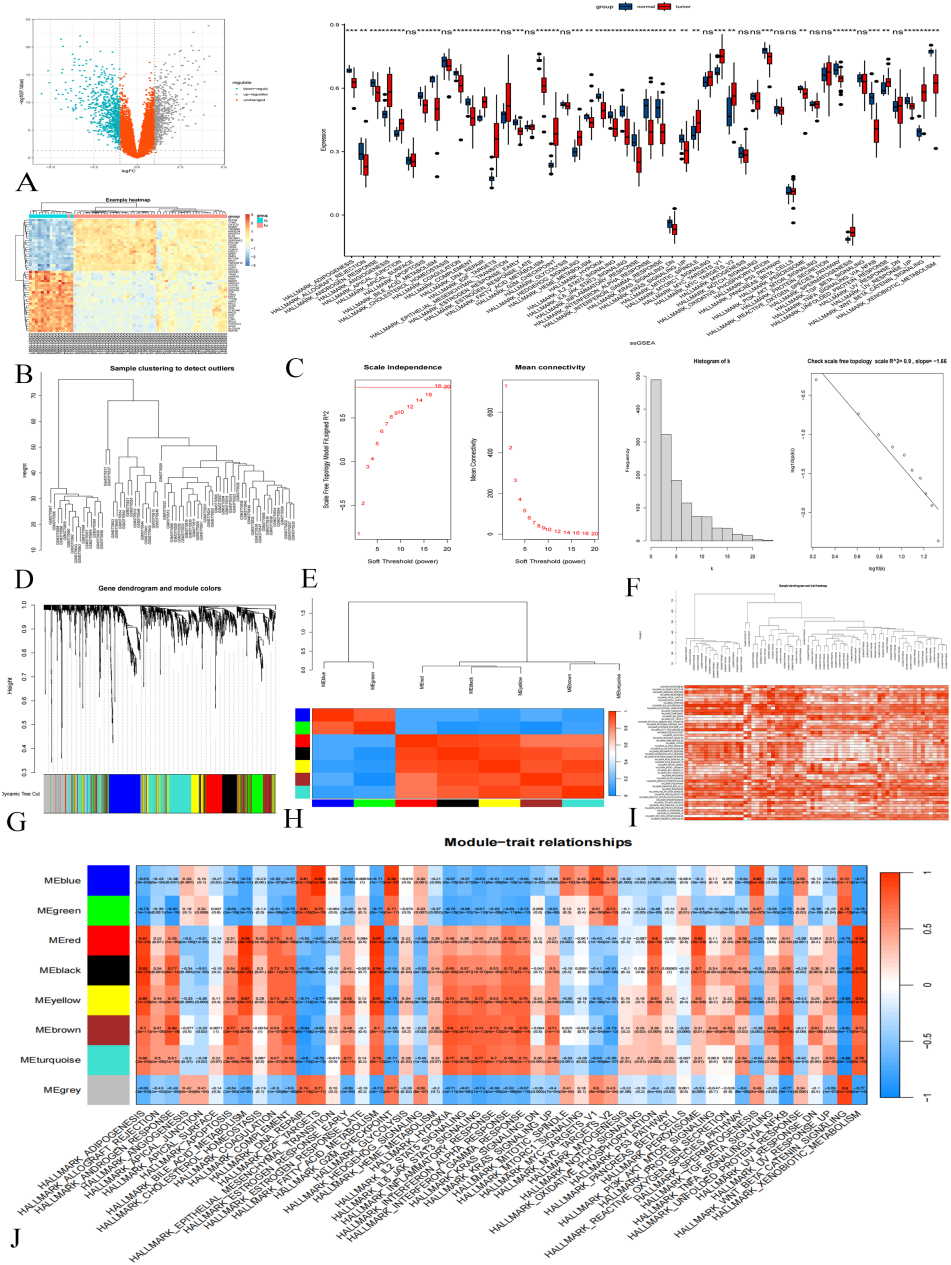
Difference Analysis and WGCNA in the GSE131329 Dataset. **(A)** Differential analysis volcano plot; gray indicates upregulated genes, downregulated blue, and orange not statistically significant. **(B)** Cluster heat map showing gene expression differences. **(C)** ssGSEA results; p < 0.001 marked ***, p < 0.01 as **, p < 0.05 as *, and p > 0.05 as ns. **(D)** Sample clustering diagram. **(E)** Soft threshold determination. **(F)** Histograms and logarithmic plots. **(G)** Gene clustering tree. **(H)** Module correlation heat map. **(I)** Sample clustering and gene set enrichment heat map. **(J)** Heat map of gene module-gene set relationships.

Difference analysis of training group data set GSE131329 identified 1377 DEGs (**Figures 1A, B**). ssGSEA analysis showed that certain classical gene sets were significantly up-regulated or down-regulated in tumor samples (**Figure 1C**).

Next, WGCNA was conducted to analyze the expression patterns of these DEGs. The clustering analysis of 67 samples yielded satisfactory results (**Figure 1D**) without the need for further optimization. Setting the soft threshold to 18 achieved a topological model fitting degree of 0.85, indicating a scale-free property (**Figure 1E**). The connection degree distribution histogram and logarithmic plot approximated linearity, further validating the scale-free characteristics of our co-expression network (**Figure 1F**). Clustering tree analysis revealed a high concentration of genes in the blue module, while unclassified genes were grouped in gray (**Figure 1G**). Correlation analysis identified seven gene modules with significant inter-module correlations (**Figure 1H**).

To highlight differences in gene set activity, we employed color coding to represent enrichment scores. Samples exhibited consistently high scores (dark red) or low scores (white), indicating significant variations in gene set activity across samples (**Figure 1I**). In the WGCNA analysis, gene set enrichment scores served as clinical features, elucidating relationships between modular genes and specific gene sets. We selected 558 genes from the colored modules (blue, green, red, black, yellow, brown) for further analysis (**Figure 1J**).

### Difference analysis results of GSE133039 and WGCNA

**Figure 2:**
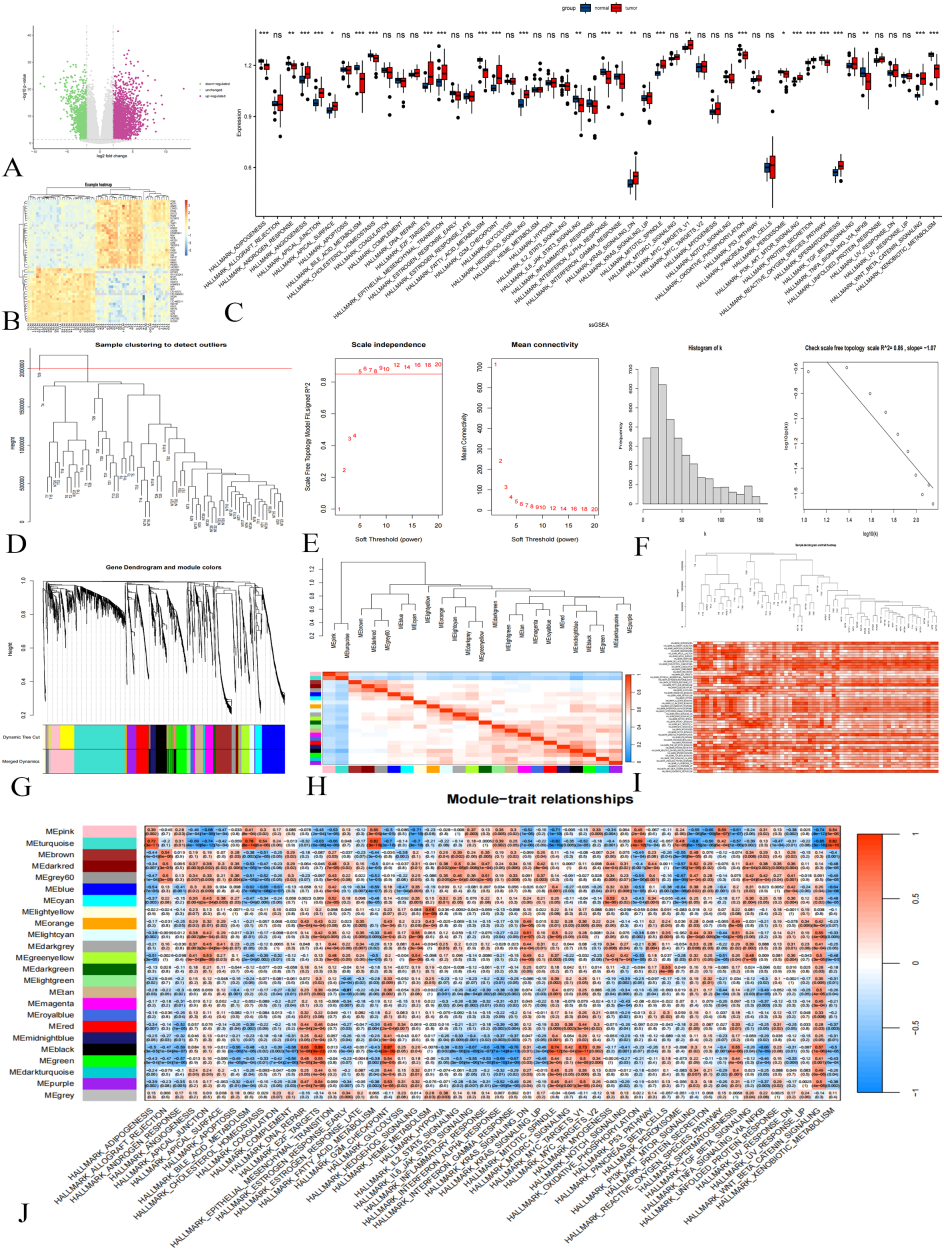
Difference Analysis and WGCNA in the GSE133039 Dataset. **(A)**: Differential analysis volcano plot; purple indicates upregulated genes, green downregulated, and gray not statistically significant. **(B)**: Cluster heat map showing gene expression differences. **(C)**: ssGSEA results; p < 0.001 marked ***, p < 0.01 as **, p < 0.05 as *, and p > 0.05 as ns. **(D)**: Sample clustering diagram. **(E)**: Soft threshold determination. **(F)**: Histograms and logarithmic plots. **(G)**: Gene clustering tree. **(H)**: Module correlation heat map. **(I)**: Sample clustering and gene set enrichment heat map. **(J)**: Heat map of gene module-gene set activity relationships.

A total of 1,216 differential genes were identified through the analysis and validation of the dataset GSE133039 (**Figures 2A, B**). The results of the ssGSEA analysis are presented in **Figure 2C**.

Initially, sample clustering analysis was performed on a dataset comprising 63 samples. After removing one outlier, 62 samples were retained for subsequent analyses (**Figure 2D**). As shown in **Figure 2E**, a soft threshold of 5 resulted in a fitting degree of the topological model reaching 0.85, indicating that the network exhibits scale-free characteristics. The histograms and logarithmic plots further verified that the constructed gene co-expression network possesses scale-free properties (**Figure 2F**).

Gene cluster tree analysis revealed the presence of multiple gene modules, prompting the combination of modules with a correlation of module feature vectors greater than 0.75 (**Figure 2G**). Module eigenvector correlation analysis yielded a total of 23 gene modules along with one gray module (**Figure 2H**). The enrichment of the 62 samples with respect to the gene set is illustrated in **Figure 2I**. Additionally, a heatmap depicting the relationships between gene modules and gene set activity is shown in **Figure 2J**. For further analysis, we selected genes from the turquoise and black modules, totaling 1,049 genes.

### PPI network construction and enrichment analysis

**Figure 3:**
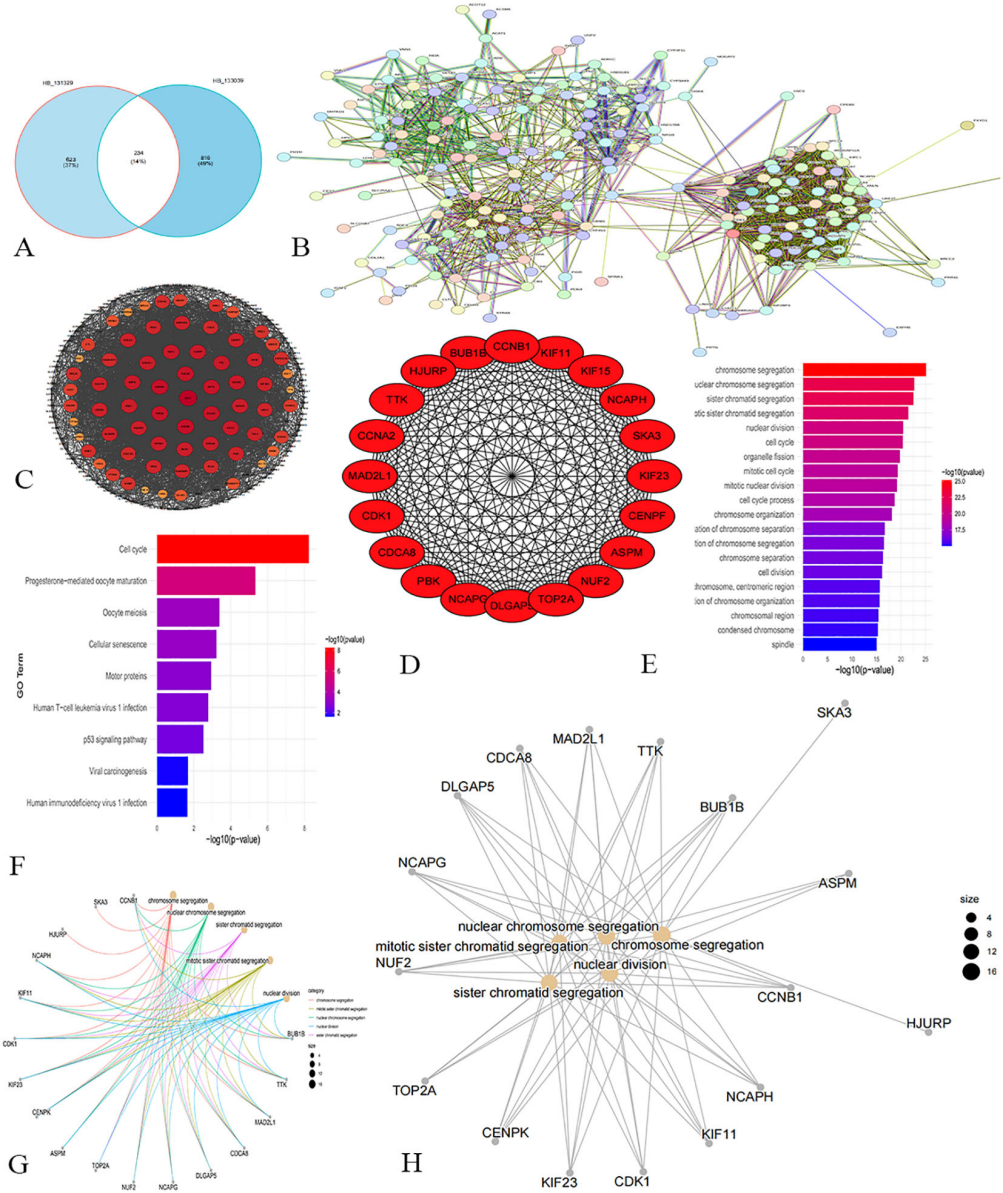
Screening and analysis results of hub gene. **(A)** Venn diagram. **(B)** PPI network analysis reveals the interaction relationship between proteins. **(C)** Network chart shows the higher degree of gene network. **(D)** Network diagram of the top 20 Hub genes. **(E)** The resulting map of GO enrichment analysis. **(F)** The result map of KEGG enrichment analysis. **(G, H)** Enrichment analysis network maps show that these Hub genes influence multiple cell functions and potential biological roles.

The Venn diagram (**Figure 3A**) showed that there were 234 common genes in the WGCNA results of the training group and the verification group. Then PPI network analysis on these genes, visualizing the results using Cytoscape (**Figure 3B**). During visualization, we excluded less connected genes to streamline the network (**Figure 3C**). We calculated the degree value of each gene and identified the top 20 Hub genes with the highest degree using the CytoHubba plug-in (**Table 1**). The Hub genes include PBK, BUB1B, TTK, MAD2L1, CDCA8, DLGAP5, NCAPG, NUF2, TOP2A, ASPM, CENPK, KIF23, CDK1, KIF11, NCAPH, CCNA2, HJURP, SKA3, KIF15, and CCNB1 (**Figure 3D**).

**Table 1:** Network topological characteristic of top 20 nodes in PPI network.

Gene	Degree	Average Shortest Path Length	Betweenness Centrality	Closeness Centrality	Clustering Coefficient	Stress	Topological Coefficient
TTK	122	2.37	0.039117218	0.421940928	0.882513661	15902	0.60709782
NCAPH	118	2.665	0.000188949	0.375234522	0.937463472	356	0.708474576
DLGAP5	118	2.665	0.000188949	0.375234522	0.937463472	356	0.708474576
NCAPG	120	2.66	0.001643118	0.37593985	0.907344633	678	0.6975
CCNB1	126	2.47	0.013081708	0.4048583	0.83922171	9192	0.626245847
KIF23	122	2.655	0.003945458	0.376647834	0.883060109	3730	0.688114754
KIF11	124	2.475	0.029108234	0.404040404	0.861448969	8784	0.640986717
CENPK	106	2.695	0.000136127	0.371057514	0.953555878	266	0.716745283
SKA3	120	2.66	0.000558842	0.37593985	0.911864407	576	0.699166667
KIF15	118	2.665	0.000188949	0.375234522	0.937463472	356	0.708474576
HJURP	122	2.595	0.005479776	0.385356455	0.887978142	2694	0.682048168
MAD2L1	120	2.66	0.001186715	0.37593985	0.907909605	648	0.6975
CCNA2	124	2.475	0.013549124	0.404040404	0.86197779	9178	0.634846212
CDCA8	120	2.6	0.001063586	0.384615385	0.917514124	1776	0.693209877
BUB1B	120	2.6	0.001063586	0.384615385	0.917514124	1776	0.693209877
PBK	118	2.665	0.000188949	0.375234522	0.937463472	356	0.708474576
CDK1	134	2.25	0.070285848	0.444444444	0.748982361	32428	0.488272921
TOP2A	124	2.34	0.043594258	0.427350427	0.86250661	20956	0.548387097
NUF2	122	2.595	0.001542318	0.385356455	0.892896175	2032	0.684274438
ASPM	122	2.625	0.006810228	0.380952381	0.882513661	1770	0.671531387

TTK, TTK Protein Kinase; NCAPH, Non-SMC Condensin I Complex Subunit H; DLGAP5, DLG Associated Protein 5; NCAPG, Non-SMC Condensin I Complex Subunit G; CCNB1, Cyclin B1; KIF23, Kinesin Family Member 23; KIF11, Kinesin Family Member 11; CENPK, Centromere Protein K; SKA3, Spindle and Kinetochore Associated Complex Subunit 3; KIF15, Kinesin Family Member 15; HJURP, Holliday Junction Recognition Protein; MAD2L1, Mitotic Arrest Deficient 2 Like 1; CCNA2, Cyclin A2; CDCA8, Cell Division Cycle Associated 8; BUB1B, BUB1; Mitotic Checkpoint Serine/Threonine; PBK, PDZ Binding Kinase B; CDK1, Cyclin Dependent Kinase 1; TOP2A, DNA Topoisomerase II Alpha; NUF2, NUF2 Component of NDC80 Kinetochore Complex; ASPM, Abnormal Spindle Microtubule Assembly.

GO enrichment analysis (**Figure 3E**) revealed that the core genes are significantly associated with biological processes related to chromosomes and the cell cycle, including chromosome segregation, nuclear division, and cell cycle regulation. These findings highlight the critical role of these 20 genes in essential cellular processes. KEGG pathway analysis indicated involvement in pathways like the cell cycle, progesterone-mediated oocyte maturation, and oocyte meiosis (**Figure 3F**), reflecting the genes’ roles in cell cycle regulation, reproductive biology, cell aging, and tumor suppression. **Figures 3G, H** illustrate that the biological functions of these core genes focus on chromosome segregation, mitotic sister chromatid segregation, and nuclear division, underscoring their importance in cell division and genetic material transfer.

### Construct machine learning algorithm model and ROC curve

**Figure 4:**
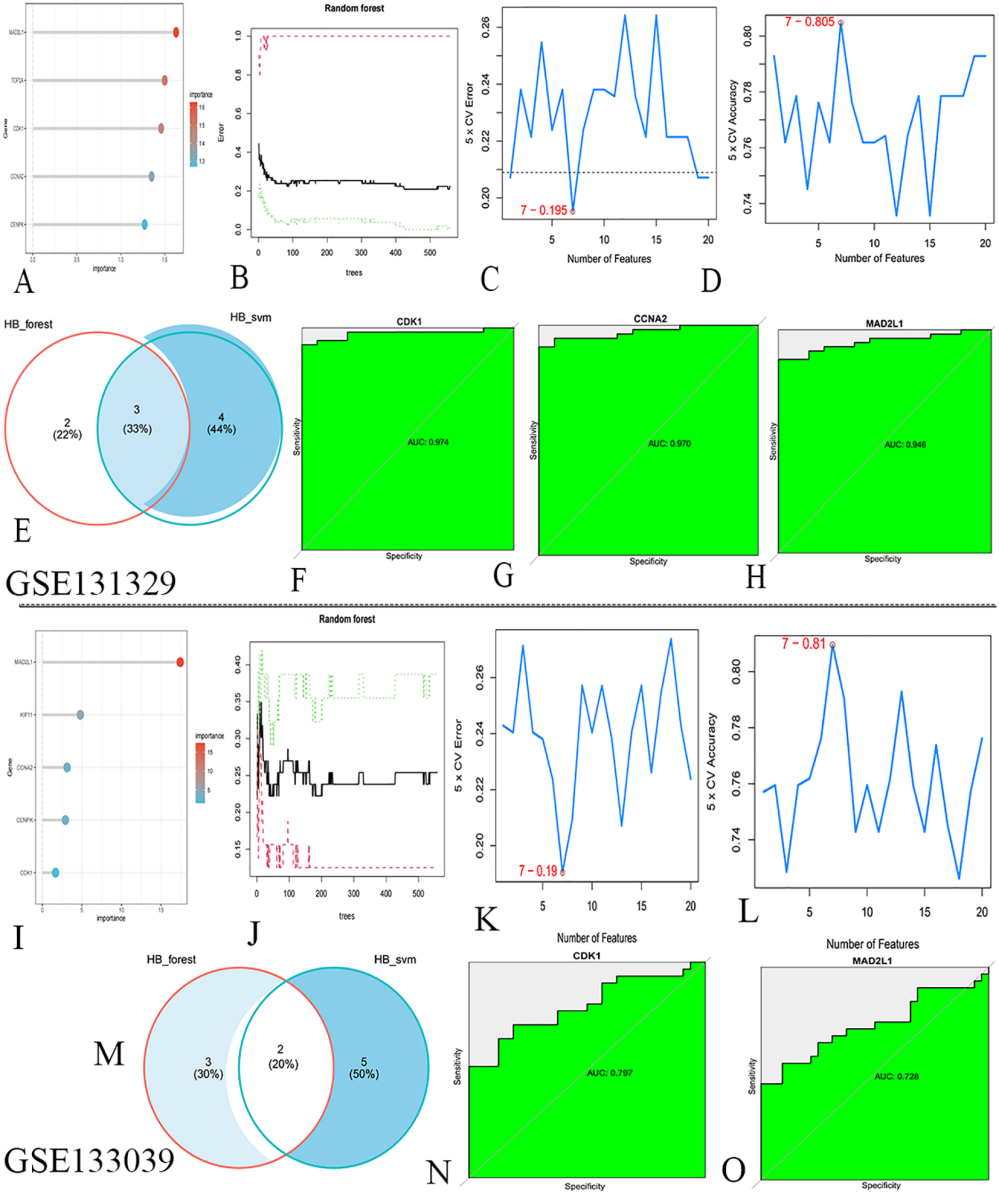
Machine learning algorithms screen for key genes. **(A)** RF identified HB biomarkers in GSE131329. **(B)** Top five genes from RF. **(C)** SVM-RFE selected seven genes, achieving a minimum error rate of 0.195. **(D)** Seven genes from SVM-RFE with 0.805 accuracy. **(E)** Venn diagram. **(F–H)** ROC curve analysis for key genes. **(I)** RF identified HB biomarkers in GSE133039. **(J)** Top five genes from RF. **(K)** SVM-RFE selected seven genes, with a minimum error rate of 0.19. **(L)** Seven genes from SVM-RFE, achieving 0.81 accuracy. **(M)** Venn diagram. **(N, O)** ROC curve analysis for key genes.

In the GSE131329 dataset, we developed RF and SVM-RFE models for HB using the expression matrix of 20 hub genes. The top five diagnostic genes identified in the RF model were CDK1, MAD2L1, CCNA2, TOP2A, and CENPK (**Figures 4A, B**). The SVM-RFE model, optimal with seven genes, achieved a minimum error rate of 0.195 and a maximum accuracy of 0.805 (**Figures 4C, D**), selecting CDK1, MAD2L1, CCNA2, CCNB1, KIF11, HJURP, and NCAPH. A Venn diagram revealed three overlapping key genes: CDK1, CCNA2, and MAD2L1 (**Figure 4E**). The AUC for the CDK1was 0.974(**Figure 4F**), with sensitivity at 0.925, specificity at 0.95. The AUC for CCNA2 was 0.970 (**Figure 4G**), with sensitivity at 0.905, specificity at 0.930. The AUC for the MAD2L1 was 0.946 (**Figure 4H**), with sensitivity at 0.868, specificity at 0.915.

In the validation dataset GSE133039, the RF model again identified CDK1, MAD2L1, CCNA2, KIF11, and CENPK as the top five genes (**Figures 4I, J**). The SVM-RFE model also performed best with seven genes, yielding an error rate of 0.19 and accuracy of 0.81 (**Figures 4K, L**), with selected genes including CDK1, MAD2L1, TOP2A, PBK, TTK, KIF15, and HJURP. A Venn diagram highlighted two co-existing key genes: CDK1 and MAD2L1 (**Figure 4M**). The AUC for the CDK1was 0.797 (**Figure 4N**), with sensitivity at 0.709, specificity at 0. 812. The AUC for the MAD2L1 was 0.728 (**Figure 4O**), with sensitivity at 0.848, specificity at 0. 906.The contribution of core genes in tumor diagnosis via the RF and SVM models is detailed in **Table 2**.

**Table 2:** Feature importance in random forests and support vector machine algorithms.

	GSE131329		GSE133039	
	RF	SVM	RF	SVM
Gene	Importance	Average Rank	Importance	Average Rank
CDK1	1.7	5.7	1.7	6.2
TOP2A	1.6	13.6	1.4	1.4
MAD2L1	1.6	5.1	17.4	8.9
CCNA2	1.5	3.2	3.1	16.3
CENPK	1.3	14	2.9	17
KIF11	1.3	4.5	4.8	11.5
CDCA8	1.1	15.9	1.0	11.5
KIF15	1.1	16.6	1.0	5.5
CCNB1	1.0	6.3	1.5	13.9
NCAPG	1.0	13.7	1.2	17
BUB1B	1.0	11.9	1.1	16.6
SKA3	0.9	14.9	1.1	10.6
DLGAP5	0.9	16.2	1.0	10.6
PBK	0.9	13.2	0.9	3.6
NUF2	0.9	7.9	0.8	9.4
ASPM	0.8	14	0.8	10.2
HJURP	0.9	4.7	0.7	6
NCAPH	0.8	7.5	0.6	14.4
KIF23	0.7	10.5	0.6	14.2
TTK	0.5	16.6	0.5	5.2

RF, Random Forest; RF, Support Vector Machine.

### Validation of MAD2L1 as a biomarker of HB

CDK1 and CCNA2 have excellent performance in AUC value, sensitivity and specificity and have been identified as biomarkers in hepatoblastoma (32–35), so their functions and roles have been relatively clear. Although MAD2L1 has been studied in other types of cancer (36–39), its role in hepatoblastoma has not been fully explored, Moreover, MAD2L1 has good performance in AUC value, sensitivity and specificity, indicating that it has high diagnostic value. So MAD2L1 was selected for further functional validation to determine its feasibility as a potential biomarker for hepatoblastoma.

**Figure 5:**
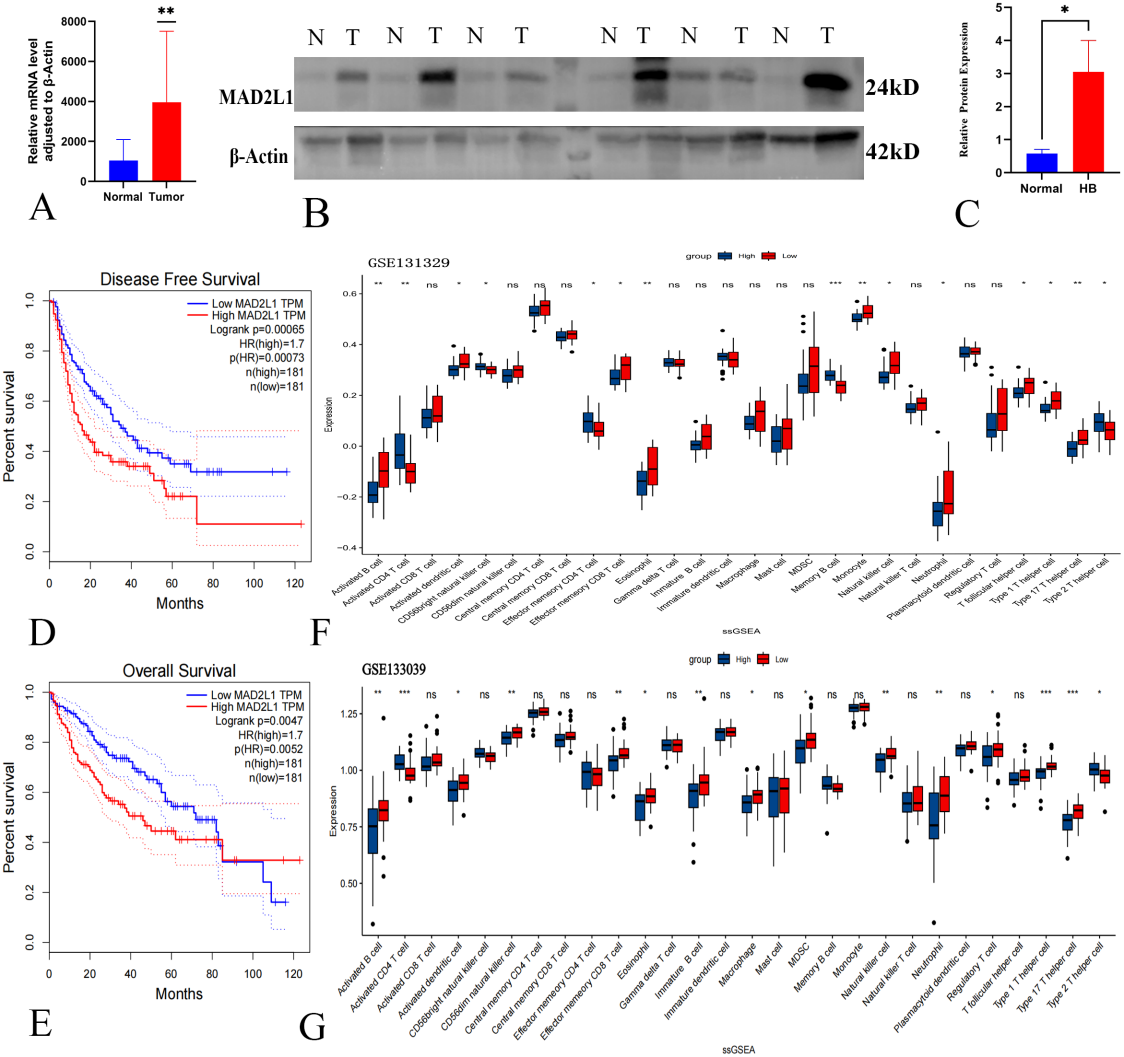
Validation of MAD2L1 as a biomarker of HB. **(A)** qPCR detected MAD2L1 mRNA levels in HB tumor and adjacent normal tissues. **(B)** WB analysis showed MAD2L1 protein expression in HB tumors (T) vs. non-cancerous tissues (N). **(C)** Quantitative analysis of MAD2L1 in HB (n = 6). **(D)** Kaplan-Meier analysis of MAD2L1 expression and DFS in HB patients. **(E)** OS analysis of MAD2L1 expression in HB patients. **(F, G)** MAD2L1 ssGSEA analysis of immune cell infiltration in GSE131329 and GSE133039 datasets. *p < 0.05, **p < 0.01, ***p < 0.001.

We analyzed MAD2L1 expression in six surgically resected HB tumor tissues and adjacent normal liver tissues. PCR results indicated that MAD2L1 mRNA levels were significantly elevated in HB tumors (**Figure 5A**), which was corroborated by Western blotting showing increased protein levels (**Figures 5B, C**).

Kaplan-Meier survival analysis revealed that high MAD2L1 expression correlated with poorer disease-free survival and overall survival in HB patients (P < 0.05) (**Figures 5D, E**), suggesting its potential role in prognosis. Additionally, ssGSEA analysis of the GSE131329 dataset showed significant increases in activated CD4 T cells, CD56 bright natural killer cells, and other immune cells in the high MAD2L1 expression group (**Figure 5F**). A similar trend was observed in the GSE133039 dataset (**Figure 5G**). These immune cells play a key role in the anti-tumor response, influencing patient prognosis by inhibiting tumor cell proliferation and reducing the risk of metastasis. The results of ssGSEA analysis further suggest the potential value of MAD2L1 as a prognostic marker in patients with hepatoblastoma.

### MAD2L1 promotes the proliferation of HB cell lines

**Figure 6:**
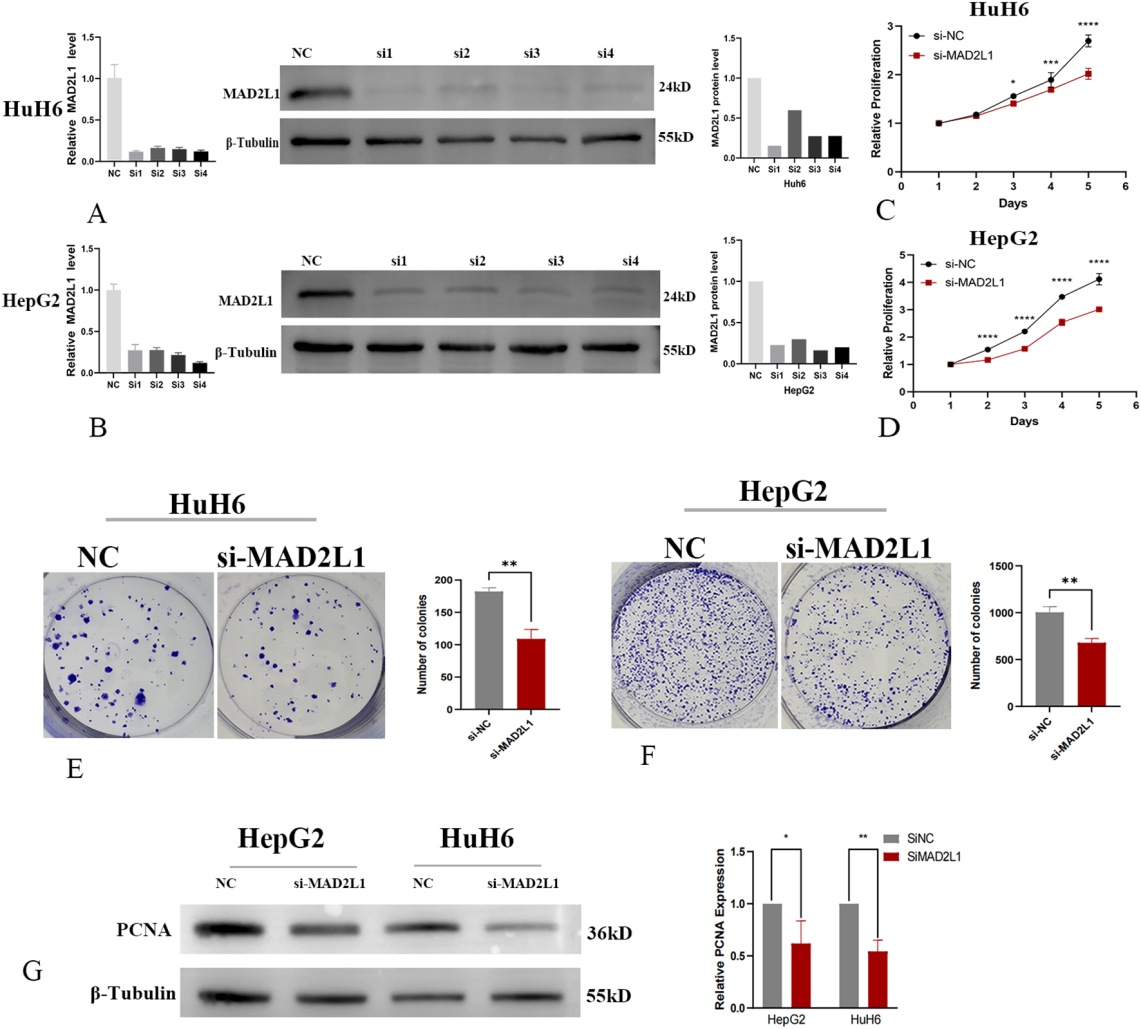
MAD2L1 promotes hepatoblastoma cell proliferation. **(A, B)** HuH6 and HepG2 cells were transfected with NC or four siRNAs targeting MAD2L1; relative mRNA levels were assessed by RT-qPCR. Knockdown efficiency evaluated by Western blot. **(C, D)** CCK-8 assay assessed proliferation effects. **(E, F)** The effect of MAD2L1 knockdown on cell proliferation was explored using a colony formation assay. **(G)** The effect of MAD2L1 knockdown on PCNA expressions was detected by western blotting. *p < 0.05, **p < 0.01, ***p < 0.001, p ****<0.0001.

In this study, we investigated the effect of MAD2L1 knockdown on the proliferation capacities of HB cell lines HuH6 and HepG2. Using siRNA, we successfully downregulated MAD2L1 expression at both the mRNA and protein levels, as confirmed by PCR and Western blot analyses (**Figures 6A, B**). The CCK-8 assay (**Figures 6C, D**) and colony formation assay (**Figures 6E, F**) revealed a significant reduction in proliferation in the MAD2L1-siRNA group compared to the control group (P < 0.05). PCNA was commonly used as markers of tumor cell proliferation (40, 41). As shown in **Figure 6G**, MAD2L1 knockdown significantly decreased the expression levels of proliferative marker PCNA, providing further evidence that silencing MAD2L1 markedly inhibits the proliferation of HuH6 and HepG2 cell lines.

### MAD2L1 promotes migrative and invasive capacities of HB cell lines

**Figure 7:**
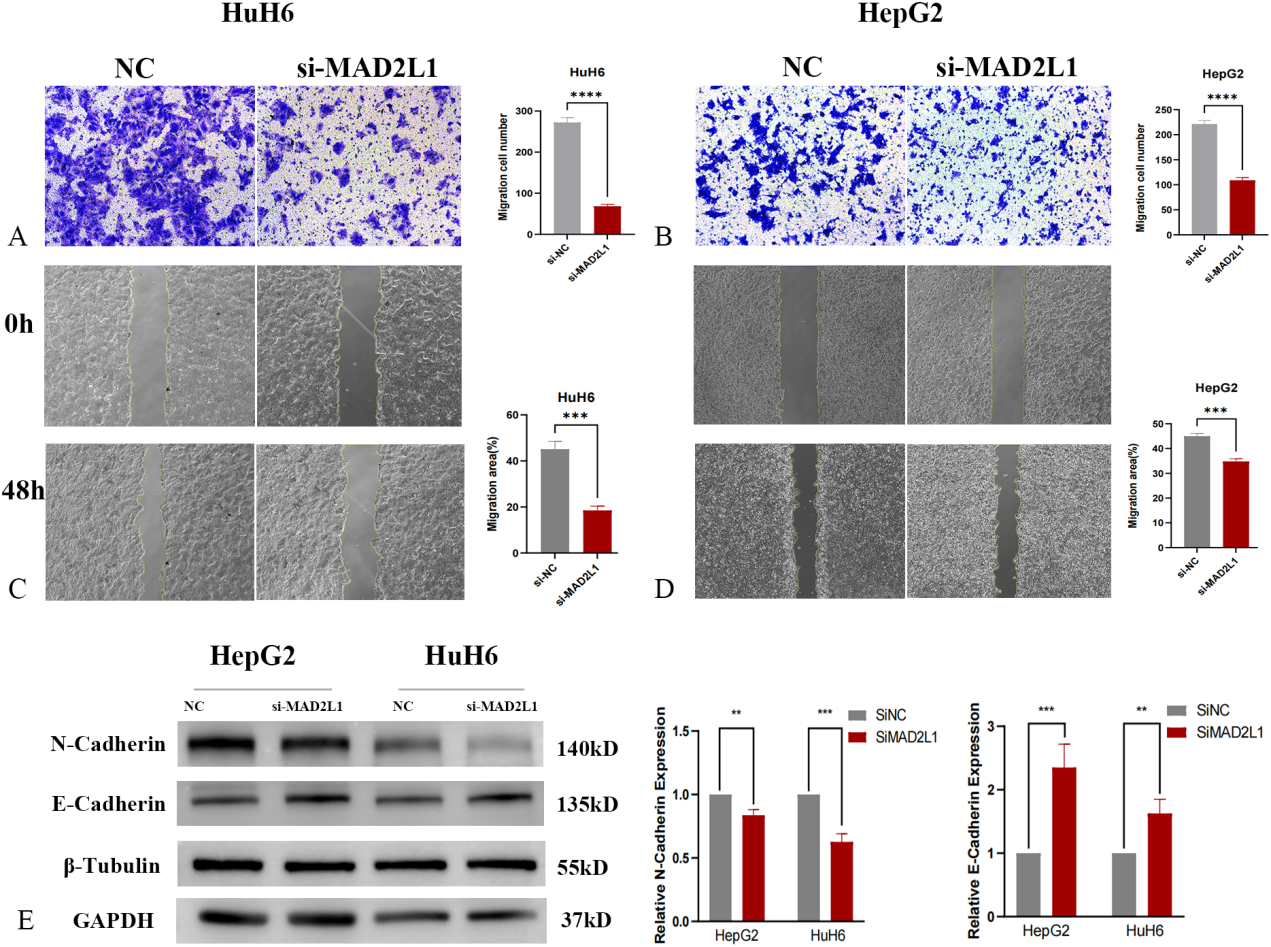
MAD2L1 promotes hepatoblastoma cell migration and invasion. **(A, B)** Transwell assays evaluated invasion capability. **(C, D)** Scratch assays assessed migration effects. **(E)** The expression levels of EMT markers were determined by western blotting. *p < 0.05, **p < 0.01, ***p < 0.001, p ****<0.0001.

In this study, we investigated the effect of MAD2L1 knockdown on the migration and invasion abilities of HB cell lines HuH6 and HepG2. Transwell invasion assays demonstrated a significant reduction in the invasion rate in the MAD2L1 knockdown group compared to the control group (**Figures 7A, B**). Similarly, scratch assays revealed that the migration distance was significantly reduced in the MAD2L1 knockdown group (**Figures 7C, D**). Epithelial-mesenchymal transition (EMT) markers, which play a crucial role in malignant tumor progression and are closely associated with aggressive behaviors such as invasion and migration (42, 43), were also analyzed. As shown in **Figure 7E**, MAD2L1 knockdown decreased N-cadherin expression (a mesenchymal marker) and increased E-cadherin expression (an epithelial marker) in both HuH6 and HepG2 cells. These findings suggest that MAD2L1 knockdown significantly inhibits the migrative and invasive capacities of HuH6 and HepG2 cell lines, potentially by regulating the EMT process. This highlights the critical role of MAD2L1 in hepatoblastoma progression.

### MAD2L1 promotes cell cycle progression by regulating E2F

In the WGCNA results of dataset GSE131329, MAD2L1 is enriched in the blue module. This module is mainly closely associated with the following gene sets: E2F_TRANSITION, G2M_CHECKPOINT. In WGCNA analysis of dataset GSE133039, MAD2L1 is enriched in black modules. This module is also mainly related to E2F_TARGETS and G2M_CHECKPOINT.

**Figure 8:**
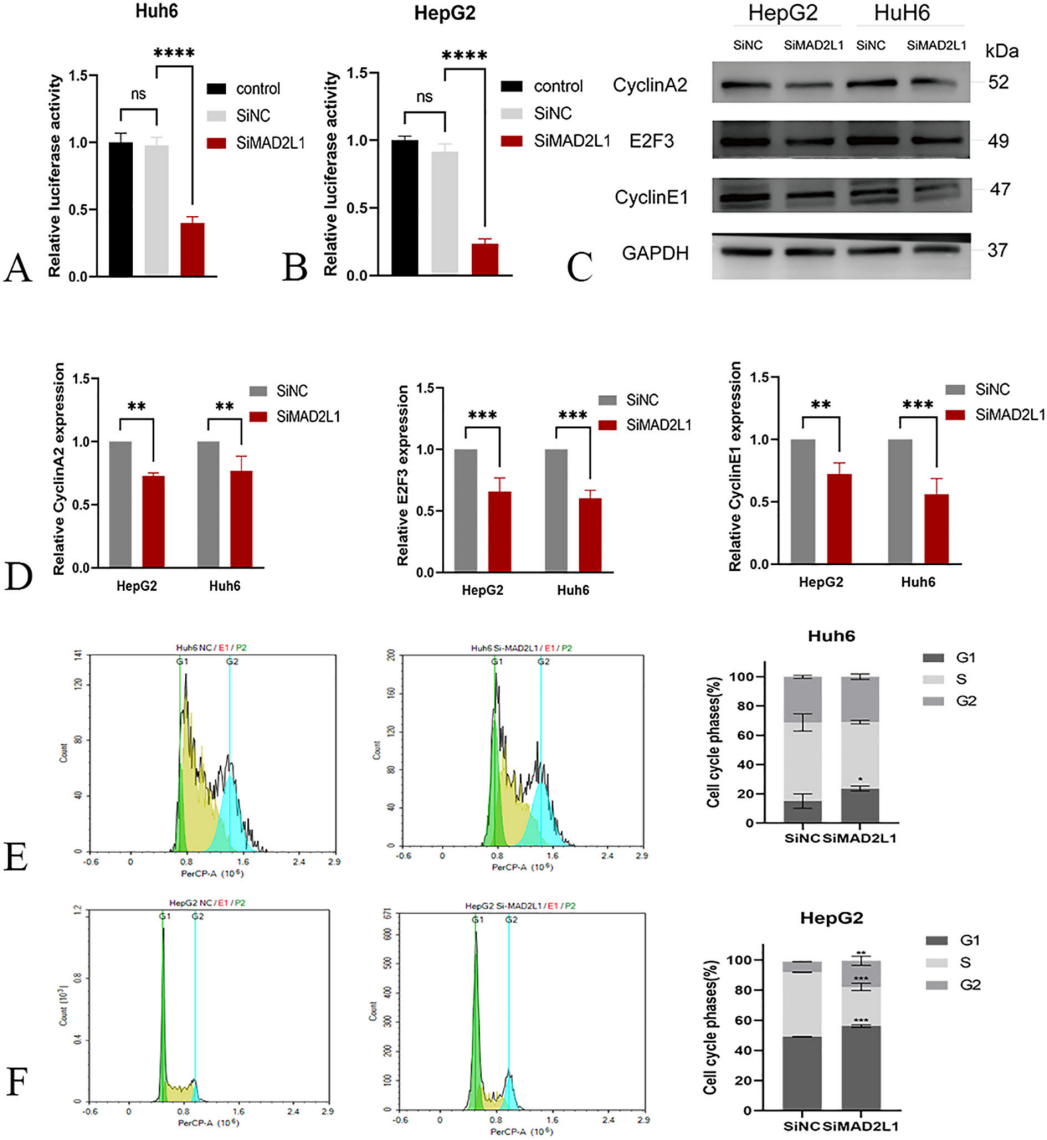
MAD2L1 activates E2F transcription and regulates cell cycle regulators. **(A, B)** E2F transcription factor relative luciferase activity in dual-luciferase reporter gene assay. **(C, D)** The effect of MAD2L1 knockdown on Cyclin A2, E2F3 and Cyclin E1 expressions was detected by western blotting. **(E, F)** The effect of MAD2L1 knockdown on cell cycle was explored using a flow cytometry assay. *p < 0.05, **p < 0.01, ***p < 0.001, p ****<0.0001.

To further verify the regulatory effect of MAD2L1 on E2F, we first used the dual-luciferase reporter assay to measure the relative luciferase activity of hepatoblastoma cells co-transfected with siRNA and specific luciferase reporter plasmids. The results showed that compared with the control group, the luciferase activity was significantly decreased after MAD2L1 was silenced (p<0.001) (**Figures 8A, B**), which proved that MAD2L1 activated the transcription of the E2F family. Subsequently, Western blot was used to detect changes in protein levels of E2F3 and its downstream target genes (Cyclin A2 and Cyclin E1), and the results showed that the expression levels of these proteins decreased after MAD2L1 was silenced (**Figures 8C, D**). This suggests that MAD2L1 may promote the expression of cell cycle related genes by activating transcription of E2F3. To further investigate the potential mechanism by which MAD2L1 promotes tumor cell proliferation, we analyzed cell cycle distribution using flow cytometry. The results of PI staining showed that after MAD2L1 silencing, the proportion of S-phase cells decreased, while the proportion of G1 phase cells increased significantly, and G1 phase block appeared (**Figures 8E, F**). It was further shown that MAD2L1 promotes the cell to enter the S phase by activating E2F3, thus speeding up the cell cycle process.

## Discussion

Bioinformatics is an integrated field that combines computer science and biology to analyze various data types (44, 45). Machine learning identifies data patterns by simulating human learning and employs techniques like support vector machines, random forests, and logistic regression to enhance performance (46, 47). The integration of machine learning into bioinformatics frameworks has improved prediction interpretability and reproducibility (48). To our knowledge, the association between gene set activity and WGCNA and HB, as well as validation of MAD2L1 in hepatoblastoma, are the first reports.

Using bioinformatics analysis and machine learning algorithms, we finally identified three key genes with the most significant diagnostic value: CDK1, CCNA2, and MAD2L1. CDK1 and CCNA2 as biomarkers of HB have been verified in studies (49–51), demonstrating the effectiveness and repeatability of our use of machine learning combined with biological information to screen biomarkers of HB. Our study reveals significant differential expressions of MAD2L1 in tumors, suggesting that it may play a distinct role in the development and progression of HB. In addition, the results of survival analysis showed that the expression level of MAD2L1 was significantly correlated with the prognosis of HB (p < 0.05). Immunoinfiltration analysis further revealed that the high expression of MAD2L1 was associated with a significant increase in the level of immune cell infiltration, suggesting that MAD2L1 may be associated with a stronger anti-tumor immune response, which may improve patient prognosis. These findings provide an important basis for confirming MAD2L1 as a biomarker for the diagnosis and prognosis of hepatoblastoma.

In our study, we used ssGSEA to assess the enrichment scores of gene sets, combined with WGCNA methods, to explore the relationships between samples and their association with gene set activity through cluster analysis and heat map visualization. Then the gene modules and key genes closely related to gene sets can be identified effectively. The quantification of gene set enrichment activity in this study provides information for cell and tissue function, contributing to insight into complex biological processes and thereby revealing disease mechanisms (29, 52). WGCNA analysis showed that MAD2L1 is mainly closely associated with the following gene set: E2F_ TARGETS, G2M_CHECKPOINT. The E2F_TARGETS gene set is typically associated with cell cycle regulation, particularly with genes regulated by E2F transcription factors. These genes play a pivotal role in the transition from the G1 to the S phase of the cell cycle (53, 54). The G2M_CHECKPOINT gene set is involved in regulating the progression of cells from the G2 phase to mitosis (M phase) and serves as a crucial mechanism in cell division and cell cycle control (55, 56). This suggests the importance of MAD2L1 in cell cycle regulation in hepatoblastoma, particularly during DNA replication and cell division. This finding is not only consistent with the enrichment results of chromosome separation and cell proliferation-related functions in the GO analysis but also reflects the potential importance of cell cycle-related pathways in the KEGG analysis.

The experimental results demonstrate that MAD2L1 knockdown significantly inhibits the proliferation, migration, and invasion of HB cell lines. In addition, we confirm that MAD2L1 promotes cell cycle progression, particularly the G1/S phase transition, by activating E2F3, thus accelerating cell proliferation. This provides new evidence for its role in tumor progression. HB, a highly proliferative hepatic malignancy. Our findings suggest that this could be linked to the activation of downstream cell cycle gene expression by MAD2L1, which enhances cell proliferation and bypasses mitotic checkpoint abnormalities, thereby helping HB cells maintain stability and promoting tumor growth. In conclusion, this study confirms the pivotal role of MAD2L1 in the proliferation and biological characteristics of cancer cells.

MAD2L1 exhibits significant potential as both a biomarker for early diagnosis and a therapeutic target in hepatoblastoma. Its upregulation suggests that detecting MAD2L1 expression could serve as a non-invasive diagnostic tool, potentially improving early detection and risk stratification when combined with existing markers like AFP. Moreover, the development of targeted therapies against MAD2L1, including small molecule inhibitors, RNA interference could provide new treatment strategies, particularly for patients with poor prognosis. Given its role in cell cycle regulation, MAD2L1 inhibition may enhance chemotherapy sensitivity or be leveraged in synthetic lethality approaches. To translate these findings into clinical practice, future research should prioritize the validation of MAD2L1-based diagnostic tests in prospective clinical trials and assess the feasibility of targeted therapies in preclinical drug development. Establishing MAD2L1 as a clinically actionable target could lead to personalized treatment strategies and improve outcomes for hepatoblastoma patients. Therefore, future studies should be combined with *in vivo* experiments to observe the effect of MAD2L1and potential therapeutic value on tumor progression in animal models. At the same time, it is still necessary to combine larger and multi-center data sets to further validate the findings of this study in the future.

## Conclusion

This study is the first to reveal the critical role of MAD2L1 in HB. Through bioinformatics and machine learning analyses, along with *in vitro* validation, we identified MAD2L1 as a promising biomarker, particularly for its significant role in cell cycle regulation. Our findings provide new insights and evidence for the early diagnosis and mechanistic understanding of hepatoblastoma.

## Data Availability

The data presented in this study are deposited in the GEO repository and can be accessed at NCBI GEO DataSets, with the accession numbers GSE131329 and GSE133039.

## References

[1] Zsiros J Brugieres L Brock P Roebuck D Maibach R Zimmermann A. Dose-dense cisplatin-based chemotherapy and surgery for children with high-risk hepatoblastoma (SIOPEL-4): a prospective, single-arm, feasibility study. Lancet Oncol. (2013) 14:834–42. doi: 10.1016/S1470-2045(13)70272-9 PMC3730732 23831416

[2] Spector LG Birch J. The epidemiology of HB. Pediatr Blood Cancer. (2012) 59:776–9. doi: 10.1002/pbc.24215 22692949

[3] Czauderna P Lopez-Terrada D Hiyama E Häberle B Malogolowkin MH Meyers RL. Hepatoblastoma state of the art: pathology, genetics, risk stratification, and chemotherapy. Curr Opin Pediatr. (2014) 26:19–28. doi: 10.1097/MOP.0000000000000046 24322718

[4] Hooks KB Audoux J Fazli H Lesjean S Ernault T Dugot-Senant N. New insights into diagnosis and therapeutic options for proliferative hepatoblastoma. Hepatology. (2018) 68:89–102. doi: 10.1002/hep.29672 29152775

[5] Perkins JL Chen Y Harris A Diller L Stovall M Armstrong GT. Infections among long-term survivors of childhood and adolescent cancer: a report from the Childhood Cancer Survivor Study. Cancer. (2014) 120:2514–21. doi: 10.1002/cncr.28763 PMC4159255 24824782

[6] Carrillo-Reixach J Torrens L Simon-Coma M Royo L Domingo-Sàbat M Abril-Fornaguera J. Epigenetic footprint enables molecular risk stratification of hepatoblastoma with clinical implications. J Hepatol. (2020) 73:328–41. doi: 10.1016/j.jhep.2020.03.025 PMC12452110 32240714

[7] Liu W Chen S Liu B. Diagnostic and prognostic values of serum exosomal microRNA-21 in children with hepatoblastoma: a Chinese population-based study. Pediatr Surg Int. (2016) 32:1059–65. doi: 10.1007/s00383-016-3960-8 27601233

[8] Mokhlesi A Talkhabi M. Comprehensive transcriptomic analysis identifies novel regulators of lung adenocarcinoma. J Cell Commun Signal. (2020) 14:453–65. doi: 10.1007/s12079-020-00565-4 PMC7642016 32415511

[9] Talkhabi M Razavi SM Salari A. Global transcriptomic analysis of induced cardiomyocytes predicts novel regulators for direct cardiac reprogramming. J Cell Commun Signal. (2017) 11:193–204. doi: 10.1007/s12079-017-0387-5 28378126 PMC5440351

[10] Hammad A Elshaer M Tang X. Identification of potential biomarkers with colorectal cancer based on bioinformatics analysis and machine learning. MATH Biosci ENG. (2021) 18:8997–9015. doi: 10.3934/mbe.2021443 34814332

[11] Hu H Cai J Qi D Li B Yu L Wang C. Identification of potential biomarkers for group I pulmonary hypertension based on machine learning and bioinformatics analysis. Int J Mol Sci. (2023) 24:8050. doi: 10.3390/ijms24098050 37175757 PMC10178909

[12] Gholizadeh M Mazlooman SR Hadizadeh M Drozdzik M Eslami S. Detection of key mRNAs in liver tissue of hepatocellular carcinoma patients based on machine learning and bioinformatics analysis. MethodsX. (2023) 10:102021. doi: 10.1016/j.mex.2023.102021 36713306 PMC9879787

[13] Sultan G Zubair S. An ensemble of bioinformatics and machine learning approaches to identify shared breast cancer biomarkers among diverse populations. Comput Biol Chem. (2023) 108:107999. doi: 10.1016/j.compbiolchem.2023.107999 38070457

[14] Zhou Y Dong Y Sun Q Fang C. Diagnosis and prognosis of non-small cell lung cancer based on machine learning algorithms. Comb Chem High Throughput Screen. (2023) 26:2170–83. doi: 10.2174/1386207326666230110115804 36627791

[15] Liu S Zheng Q Zhang R Li T Zhan J. Construction of a combined random forest and artificial neural network diagnosis model to screening potential biomarker for hepatoblastoma. Pediatr Surg Int. (2022) 38:2023–34. doi: 10.1007/s00383-022-05255-3 36271952

[16] Wang JY Lao J Luo Y Guo JJ Cheng H Zhang HY. Integrative analysis of DNA methylation and gene expression profiling data reveals candidate methylation-regulated genes in hepatoblastoma. Int J Gen Med. (2021) 14:9419–31. doi: 10.2147/IJGM.S331178 PMC8664605 34908869

[17] Garnier A Ilmer M Becker K Häberle B von Schweinitz D Kappler R. Truncated neurokinin-1 receptor is an ubiquitous antitumor target in hepatoblastoma, and its expression is independent of tumor biology and stage. Oncol Lett. (2016) 11:870–8. doi: 10.3892/ol.2015.3951 PMC4726931 26870298

[18] Langfelder P Horvath S. WGCNA: an R package for weighted correlation network analysis. BMC Bioinf. (2008) 9:559. doi: 10.1186/1471-2105-9-559 PMC2631488 19114008

[19] Liang Y Zhang C Dai DQ. Identification of differentially expressed genes regulated by methylation in colon cancer based on bioinformatics analysis. World J Gastroenterol. (2019) 25:3392–407. doi: 10.3748/wjg.v25.i26.3392 PMC6639549 31341364

[20] Zhou Y Yang L Zhang X Chen R Chen X Tang W. Identification of potential biomarkers in glioblastoma through bioinformatic analysis and evaluating their prognostic value. BioMed Res Int. (2019) 2019:6581576. doi: 10.1155/2019/6581576 31119182 PMC6500689

[21] Xu X Zhou Y Miao R Chen W Qu K Pang Q. Transcriptional modules related to hepatocellular carcinoma survival: coexpression network analysis. Front Med. (2016) 10:183–90. doi: 10.1007/s11684-016-0440-4 27052251

[22] Zhao H Cai W Su S Zhi D Lu J Liu S. Screening genes crucial for pediatric pilocytic astrocytoma using weighted gene coexpression network analysis combined with methylation data analysis. Cancer Gene Ther. (2014) 21:448–55. doi: 10.1038/cgt.2014.49 25257306

[23] Szklarczyk D Franceschini A Wyder S Forslund K Heller D Huerta-Cepas J. STRING v10: protein-protein interaction networks, integrated over the tree of life. Nucleic Acids Res. (2015) 43:D447–52. doi: 10.1093/nar/gku1003 PMC4383874 25352553

[24] Shannon P Markiel A Ozier O Baliga NS Wang JT Ramage D. Cytoscape: a software environment for integrated models of biomolecular interaction networks. Genome Res. (2003) 13:2498–504. doi: 10.1101/gr.1239303 PMC403769 14597658

[25] Chin CH Chen SH Wu HH Ho CW Ko MT Lin CY. cytoHubba: identifying hub objects and sub-networks from complex interactome. BMC Syst Biol. (2014) 8 Suppl 4:S11. doi: 10.1186/1752-0509-8-S4-S11 25521941 PMC4290687

[26] Alazaidah R Samara G Aljaidi M Haj Qasem M Alsarhan A Alshammari M. Potential of machine learning for predicting sleep disorders: A comprehensive analysis of regression and classification models. Diagn (Basel). (2023) 14:27. doi: 10.3390/diagnostics14010027 PMC10802836 38201336

[27] Hao PY Chiang JH Chen YD. Possibilistic classification by support vector networks. Neural Netw. (2022) 149:40–56. doi: 10.1016/j.neunet.2022.02.007 35189529

[28] Houssein EH Hassan HN Samee NA Jamjoom MMA. Novel hybrid runge kutta optimizer with support vector machine on gene expression data for cancer classification. Diagn (Basel). (2023), 13 (9):1621. doi: 10.3390/diagnostics13091621 PMC10178557 37175012

[29] Subramanian A Tamayo P Mootha VK Mukherjee S Ebert BL Gillette MA. Gene set enrichment analysis: a knowledge-based approach for interpreting genome-wide expression profiles. P Natl Acad Sci USA. (2005) 102(43):15545–50. doi: 10.1073/pnas.0506580102 PMC1239896 16199517

[30] Barbie DA Tamayo P Boehm JS Kim SY Moody SE Dunn IF. Systematic RNA interference reveals that oncogenic KRAS-driven cancers require TBK1. NATURE. (2009) 462:108–12. doi: 10.1038/nature08460 PMC2783335 19847166

[31] Hänzelmann S Castelo R Guinney J. GSVA: gene set variation analysis for microarray and RNA-seq data. BMC Bioinf. (2013) 14:7. doi: 10.1186/1471-2105-14-7 PMC3618321 23323831

[32] Goga A Yang D Tward AD Morgan DO Bishop JM. Inhibition of CDK1 as a potential therapy for tumors over-expressing MYC. Nat Med. (2007) 13:820–7. doi: 10.1038/nm1606 17589519

[33] Sun R Li S Zhao K Diao M Li L. Identification of ten core hub genes as potential biomarkers and treatment target for hepatoblastoma. Front Oncol. (2021) 11:591507. doi: 10.3389/fonc.2021.591507 33868991 PMC8047669

[34] Coudreuse D Nurse P. Driving the cell cycle with a minimal CDK control network. Nature. (2010) 468:1074–9. doi: 10.1038/nature09543 21179163

[35] Aghajanzadeh T Tebbi K Talkhabi M. Identification of potential key genes and miRNAs involved in HB pathogenesis and prognosis. J Cell Commun Signal. (2020) 15:131–42. doi: 10.1007/s12079-020-00584-1 PMC7904995 33051830

[36] Wang Y Wang F He J Du J Zhang H Shi H. miR-30a-3p targets MAD2L1 and regulates proliferation of gastric cancer cells. Onco Targets Ther. (2019) 12:11313–24. doi: 10.2147/OTT.S222854 PMC6927793 31908496

[37] Gao Y Liu Y Sun L Ouyang X Zhu C Qin X. MAD2L1 functions as a novel diagnostic and predictive biomarker in cholangiocarcinoma. Genet Test Mol Biomarkers. (2021) 25:685–95. doi: 10.1089/gtmb.2021.0122 34788140

[38] Wang Z Katsaros D Shen Y Fu Y Canuto EM Benedetto C. Biological and Clinical Significance of MAD2L1 and BUB1, Genes Frequently Appearing in Expression Signatures for Breast Cancer Prognosis. PLoS One. (2015) 10 (8):e0136246. doi: 10.1371/journal.pone.013624 26287798 PMC4546117

[39] Ding X Fu Q Chen W Chen L Zeng Q Zhang S. Targeting of MAD2L1 by miR-515-5p involves the regulation of cell cycle arrest and apoptosis of colorectal cancer cells. Cell Biol Int. (2022) 46:840–8. doi: 10.1002/cbin.11774 35143103

[40] Wang SC Nakajima Y Yu YL Xia W Chen CT Yang CC. Tyrosine phosphorylation controls PCNA function through protein stability. Nat Cell Biol. (2006) 8:1359–68. doi: 10.1038/ncb1501 17115032

[41] Zhang S Zhou T Wang Z Yi F Li C Guo W. Post-translational modifications of PCNA in control of DNA synthesis and DNA damage tolerance-the implications in carcinogenesis. Int J Biol Sci. (2021) 17:4047–59. doi: 10.7150/ijbs.64628 PMC8495385 34671219

[42] Cao ZQ Wang Z Leng P. Aberrant N-cadherin expression in cancer. BioMed Pharmacother. (2019) 118:109320. doi: 10.1016/j.biopha.2019.109320 31545265

[43] Kong D Li Y Wang Z Sarkar FH. Cancer stem cells and epithelial-to-mesenchymal transition (EMT)-phenotypic cells: are they cousins or twins? Cancers (Basel). (2011) 3:716–29. doi: 10.3390/cancers30100716 PMC3106306 21643534

[44] Deng Y Wang H Hamamoto R Schaffer D Duan S. Functional genomics, genetics, and bioinformatics. BioMed Res Int. (2015) 2015:184824. doi: 10.1155/2015/184824 25977917 PMC4421104

[45] Can T. Introduction to bioinformatics. Methods Mol Biol. (2014) 1107:51–71. doi: 10.1007/978-1-62703-748-8_4 24272431

[46] LeCun Y Bengio Y Hinton G. Deep learning. Nature. (2015) 521:436–44. doi: 10.1038/nature14539 26017442

[47] Sandve GK Nekrutenko A Taylor J Hovig E. Ten simple rules for reproducible computational research. PloS Comput Biol. (2013) 9:e1003285. doi: 10.1371/journal.pcbi.1003285 24204232 PMC3812051

[48] Stodden V McNutt M Bailey DH Deelman E Gil Y Hanson B. Enhancing reproducibility for computational methods. Science. (2016) 354:1240–1. doi: 10.1126/science.aah6168 27940837

[49] Tian L Chen T Lu J Yan J Zhang Y Qin P. Integrated protein-protein interaction and weighted gene co-expression network analysis uncover three key genes in hepatoblastoma. Front Cell Dev Biol. (2021) 9:631982. doi: 10.3389/fcell.2021.631982 33718368 PMC7953069

[50] Yam CH Fung TK Poon RY. Cyclin A in cell cycle control and cancer. Cell Mol Life Sci. (2002) 59:1317–26. doi: 10.1007/s00018-002-8510-y PMC11337442 12363035

[51] Chen K You Y Tang W Tian X Zhu C Yin Z. HAND2-AS1 plays a tumor-suppressive role in hepatoblastoma through the negative regulation of CDK1. Heliyon. (2024) 10:e35930. doi: 10.1016/j.heliyon.2024.e35930 39286228 PMC11402935

[52] Liberzon A Birger C Thorvaldsdóttir H Ghandi M Mesirov JP Tamayo P. The Molecular Signatures Database (MSigDB) hallmark gene set collection. Cell Syst. (2015) 1:417–25. doi: 10.1016/j.cels.2015.12.004 PMC4707969 26771021

[53] Dyson N. The regulation of E2F by pRB-family proteins. Gene Dev. (1998) 12:2245–62. doi: 10.1101/gad.12.15.2245 9694791

[54] Bracken AP Ciro M Cocito A Helin K. E2F target genes: unraveling the biology. Trends Biochem Sci. (2004) 29:409–17. doi: 10.1016/j.tibs.2004.06.006 15362224

[55] Bartek J Lukas J. DNA damage checkpoints: from initiation to recovery or adaptation. Curr Opin Cell Biol. (2007) 19:238–45. doi: 10.1016/j.ceb.2007.02.009 17303408

[56] Kumar S Talluri S Fulciniti M Shammas M Munshi N. Elevated APEX1 disrupts G2/M checkpoint, contributing to evolution and survival of myeloma cells. BLOOD. (2015) 126:2997–7. doi: 10.1182/blood.v126.23.2997.2997

