# Supplementary material for: Identification of MAD2L1 as a novel biomarker for hepatoblastoma through bioinformatics and machine learning approaches
Source: Front Oncol. 2025 Mar 31;15:1524714. doi: 10.3389/fonc.2025.1524714 (PMC11994420; doi:10.3389/fonc.2025.1524714)
Supplement: Supplementary file 1 [file DataSheet1.docx]

**Supplementary Materials**

Data preprocessing process: For the microarray data processing (GSE131329 Affymetrix), several steps are involved. First, background correction is performed using the Robust Multi-array Average (RMA) method to correct background noise and ensure signal accuracy. Next, normalization is applied, using RMA to eliminate technical variation and ensure comparability between different samples. This process includes background correction, quantile normalization, and log-transformation. In the data filtering step, low-quality or low-expression probes are excluded, typically retaining genes that are expressed in most samples. For missing data handling, missing values are imputed using methods such as k-nearest neighbors (KNN). Finally, the expression matrix is generated for downstream analysis.

For RNA-seq data processing (GSE133039), the first step is data import, where raw data is read from FASTQ files and undergoes quality control. Then, normalization is performed using the variance stabilizing transformation (VST) method from DESeq2, which reduces technical bias, smooths the count data, and transforms it to a log scale. During data filtering, low-quality samples are excluded, and genes with more than 10% missing values are removed. Missing data handling in RNA-seq is typically addressed through gene filtering, rather than imputing missing values. Finally, the expression matrix is generated, ready for differential expression analysis and other downstream analyses.
